# Evaluation of Spousal Support and Stress Coping Styles of Pregnant Women Diagnosed with Fetal Anomaly

**DOI:** 10.3390/medicina61050868

**Published:** 2025-05-09

**Authors:** Sevim Tuncer Can, Sevler Yıldız, Ceren Sağlam, Hakan Golbasi, Atalay Ekin

**Affiliations:** 1 Department of Perinatology, Fethi Sekin City Hospital, 23280 Elazig, Turkey; sevim.tuncer@saglik.gov.tr; 2 Department of Psychiatry, Fethi Sekin City Hospital, 23280 Elazig, Turkey; 3 Department of Perinatology, İzmir City Hospital, 35170 Izmir, Turkey; ceren.saglam@saglik.gov.tr (C.S.); hakan.golbasi@saglik.gov.tr (H.G.); atalay.ekin@sbu.edu.tr (A.E.)

**Keywords:** fetal anomaly, pregnant, perceived stress, spousal support, stress-coping style

## Abstract

*Background and Objectives*: Pregnant women may experience various difficulties when abnormal conditions are detected in their babies. We examined the relationship between anxiety and depression levels, spousal support, and stress-coping styles in pregnant women diagnosed with fetal anomalies. *Materials and Methods:* A total of 157 pregnant women, 59 of whom were diagnosed with fetal anomalies and 98 of whom were healthy with no obstetric complications, were included in this study. All participants were administered the Beck Anxiety Inventory (BAI), Beck Depression Inventory (BDI), Spousal Support Scale (SSS), and Coping Styles Scale (CSS) questionnaires. The data were compared statistically. *Results:* The BAI (*p* < 0.001) and the Submissive Approach (*p* = 0.004), which is a subdimension of the CSS, were significantly higher in the group of pregnant women diagnosed with fetal anomalies than in the control group. Multivariate logistic regression analysis performed to calculate the risk of fetal anomalies showed that having a high school education or below and living in the city were associated with a higher risk of fetal anomaly than living in the countryside. The cut-off value of 4 for the BAI had a sensitivity of 64.4% and a specificity of 65.3. Additionally, a cut-off value of 6 for the Submissive Approach, a CSS subdimension, had a 66.1% sensitivity and a 57.1% specificity. A significant negative correlation was observed between the Spousal Support Scale, the BDI, and the gravidity in the case group. There was a positive correlation between the BAI and the BDI and a significant negative correlation between the BAI and these CSS subdimensions: Self-Confident Approach, Seeking Social Support, and Optimistic Approach. There was a positive correlation between the BDI and the Helpless Approach subdimension of the CSS and a significant negative correlation between the BDI and the Self-Confident Approach and Optimistic Approach subdimensions, as well as the gestational age at which fetal anomaly was detected. A significant positive correlation was observed between the BDI and the Helpless Approach subdimension of the CSS, while significant negative correlations were observed between the BDI and the Self-Confident Approach and Optimistic Approach subdimensions and the gestational age at which the fetal anomaly was detected. *Conclusions:* The pregnant women diagnosed with fetal anomalies experienced anxiety, but most tended to use the submissive coping style to deal with stress, and their partners also supported them.

## 1. Introduction

Fetal anomaly refers to a condition affecting the fetus in utero and may stem from either a physical or genetic factor, leading to both functional and structural losses. Fetal anomalies can range from minor malformations to those severe enough to result in death [1]. Recently, advances in genetic testing techniques and fetal imaging technologies have facilitated the detection of fetal anomalies [2]. The prevalence of anomalies detected via ultrasonography in the prenatal period is 2.95%, with the most frequently diagnosed fetal anomalies being those of the genitourinary system [3]. According to the International Society of Ultrasound in Obstetrics and Gynecology (ISUOG), the ideal period for screening for structural defects is the second trimester of pregnancy (18–22 weeks) [4]. Ultrasounds performed during this period are considered the gold standard for detecting structural anomalies [5].

The detection of fetal anomalies during pregnancy is associated with significant psychological symptoms in mothers [6]. Psychological distress has been noted not only in cases of severe and complex anomalies but also in less severe and treatable conditions [7]. The duration and intensity of the emotional response following a fetal anomaly diagnosis can vary, with some mothers developing depression or post-traumatic stress reactions [8]. Expectant mothers diagnosed with fetal anomalies may perceive this situation as a stressor [9]. Coping strategies are employed to mitigate the negative effects of stressful events or situations [10]. When individuals encounter threatening situations, their perception of potential loss or harm triggers coping mechanisms [11]. A relationship has been observed between experiencing and coping with negative emotions [12]. Using stress-coping strategies aims to improve self-esteem, reduce psychological symptoms, and enhance overall functionality [13]. Pregnant women diagnosed with fetal anomalies may seek support, particularly from their partners, as they navigate this stress [14]. While fathers typically play a supportive role during pregnancy, little is known about how the detection of a fetal anomaly impacts the psychological well-being of fathers [15,16]. However, psychological symptoms may emerge following the diagnosis of a fetal anomaly, potentially leading to additional mental health consequences for the mother [17].

In stressful situations, including health problems, partner support is as important as the person’s ability to cope with the stress. Both the method of coping with stress and spousal support can affect people’s mental states. If these two parameters are not at adequate levels, this may negatively affect the mental state of the person [18,19]. In summary, research examining the psychological distress endured by pregnant women following the detection of fetal anomalies is increasingly common. Our study aimed to investigate the coping styles and mental health conditions of pregnant women diagnosed with fetal anomalies, as well as the relationships between these factors, partner support, and other clinical variables. We hypothesized that stress caused by the diagnosis of fetal anomaly and the social support provided by partners influence the mental health symptoms of the expectant mother.

## 2. Materials and Methods

This study was conducted in accordance with the ethical standards outlined in the 2013 revision of the Helsinki Declaration. Ethical approval was obtained from the University of Health Sciences, Tepecik Training and Research Hospital’s Non-Interventional Clinical Research Ethics Committee (approval number 2023/09-63, dated 10 October 2023). Prior to this study, sample size calculation was conducted using G*Power software 3.1.9.2. Based on a previous study [20] reporting differences in anxiety levels between pregnant women with and without fetal anomalies (mean ± SD: 8.5 ± 4.4 vs. 5.5 ± 3.4, respectively), an effect size (Cohen’s d) of 0.76 was estimated. Using a two-tailed independent-samples *t*-test with a significance level of 0.05 and a power of 0.80, it was determined that a minimum of 27 participants in the anomaly group and 45 participants in the healthy group would be required.

The study sample consisted of women diagnosed with fetal anomalies (*n* = 70) who presented to the perinatology outpatient clinic of Izmir City Hospital between 1 December 2023 and 1 February 2024 and healthy pregnant women (*n* = 100) without known systemic or psychiatric conditions who visited the hospital for routine check-ups. These anomalies ranged from milder anomalies that could be corrected after birth to severe anomalies, such as trisomy, with a recommendation for termination of pregnancy. As approximately half of all anomalies can be detected between the 18th and 22nd weeks of pregnancy [21], we included pregnant women who visited the perinatology clinic during the second trimester in our study.

Pregnant women with fetal anomalies who voluntarily agreed to participate in this study were included, with the following inclusion criteria: were 18 years or older, were capable of answering the research questions, had no history of neurological or psychiatric disorders, and had received a fetal anomaly diagnosis at least one week prior. The control group consisted of healthy pregnant women who visited the Izmir City Hospital Department of Obstetrics and Gynecology for routine check-ups, with no known medical conditions or symptoms affecting either the mother or the fetus.

The study group was evaluated during a routine ultrasound performed by a perinatologist at the Perinatology Department of Izmir City Hospital around the 18th week of gestation due to suspected fetal malformation. The comparison group underwent a routine ultrasound at approximately 18 weeks of pregnancy. Eleven women diagnosed with fetal anomalies and two women with normal pregnancies voluntarily withdrew from this study. After obtaining of written informed consent from all participants, the following questionnaires were administered: the Sociodemographic Data Form, Spousal Support Scale (SSS), Coping Styles Scale (CSS), Beck Depression Inventory (BDI), and Beck Anxiety Inventory (BAI).

## 3. Scales Used in This Study

### 3.1. Sociodemographic and Clinical Data Form

This semi-structured form, developed by these researchers, was used to collect sociodemographic and clinical data such as gender, place of residence, disease duration, and the presence of any concomitant conditions.

### 3.2. Beck Anxiety Inventory (BAI)

This scale measures the frequency of anxiety symptoms experienced by an individual. It consists of 21 items, each rated on a Likert-type scale from 0 to 3. A higher total score indicates a greater level of anxiety. Developed by Beck et al. [22], the validity and reliability of its Turkish version were examined by Ulusoy et al. [23]. The Cronbach Alpha internal consistency score of this scale was 0.91.

### 3.3. Spousal Support Scale (SSS)

Developed by Yıldırım (2004), this scale consists of 27 items and four subdimensions (emotional support, financial aid and information support, appreciation support, and social interest support) [24]. The highest possible score is 81, and the lowest is 27. A higher score reflects a greater perceived level of spousal support [24]. The Cronbach Alpha internal consistency score of this scale was 0.90.

### 3.4. Coping Styles Scale (CSS)

Developed by Folkman and Lazarus, this scale uses a 4-point Likert-type format. The validity and reliability of the Turkish version of the 30-item form were examined by Şahin and Durak (1995) [25]. This scale includes sub-factors such as self-confident coping, helpless coping, submissive coping, optimistic coping, and seeking social support [25,26]. The internal consistency coefficient of the scale was found to be 0.86.

### 3.5. Beck Depression Inventory (BDI)

This inventory was developed by Beck to measure the risk of depression, the levels of depressive symptoms, and changes in symptom severity in adults [27]. A higher score indicates a greater severity of depressive symptoms. The validity and reliability of the Turkish version of the scale have been established [28]. The Cronbach Alpha internal consistency score of the scale was 0.87.

## 4. Statistical Analysis

The analyses were performed using SPSS (Statistical Package for the Social Sciences; SPSS Inc., Chicago, IL, USA) version 22. Descriptive data are presented as *n*, frequencies, and percentages for the categorical variables and as means ± standard deviation (Mean ± SD) and median interquartile ranges (25–75 percentile values) for the continuous variables. The comparison of the categorical variables between the groups was carried out using the Chi-square test (Pearson’s Chi-square). The normality of the continuous variables was assessed using the Kolmogorov–Smirnov test. For pairwise group comparisons, variables with normal distribution were analyzed using Student’s *t*-test and non-normally distributed variables were analyzed using the Mann–Whitney U test. The relationship between the continuous variables was evaluated using the Spearman correlation test. Logistic regression was performed to calculate the risk of high-risk pregnancy. The variables found to be statistically significant in the binary comparisons were included in a multivariate model. Receiver operating characteristic (ROC) curves were drawn to determine the possible association between scale scores and high-risk pregnancy. A *p*-value of <0.05 was considered statistically significant.

## 5. Results

A total of 157 patients were included in this study, comprising 59 women diagnosed with fetal anomalies and 98 healthy pregnant women. The proportion of university graduates in the patient group (22%) was significantly lower than in the control group (44.9%) (*p* = 0.004). The proportion of individuals living in rural areas was significantly lower in the patient group (15.3%) than in the control group (31.6%) (*p* = 0.023). In the group of women diagnosed with fetal anomalies, the BAI (*p* < 0.001) and the Submissive Approach subscale of the CSS (*p* = 0.004) were significantly higher than in the control group (Table 1).

The multivariate logistic regression analysis conducted to calculate the risk of fetal anomaly showed that having a high school education or lower increased the risk of fetal anomaly by 3.243 times (95% CI = 1.274–8.254) compared to being a university graduate. Similarly, living in the city posed a 3.054 times greater risk (95% CI = 1.090–8.560) of fetal anomaly compared with living in rural areas (Table 2).

**Figure 1:**
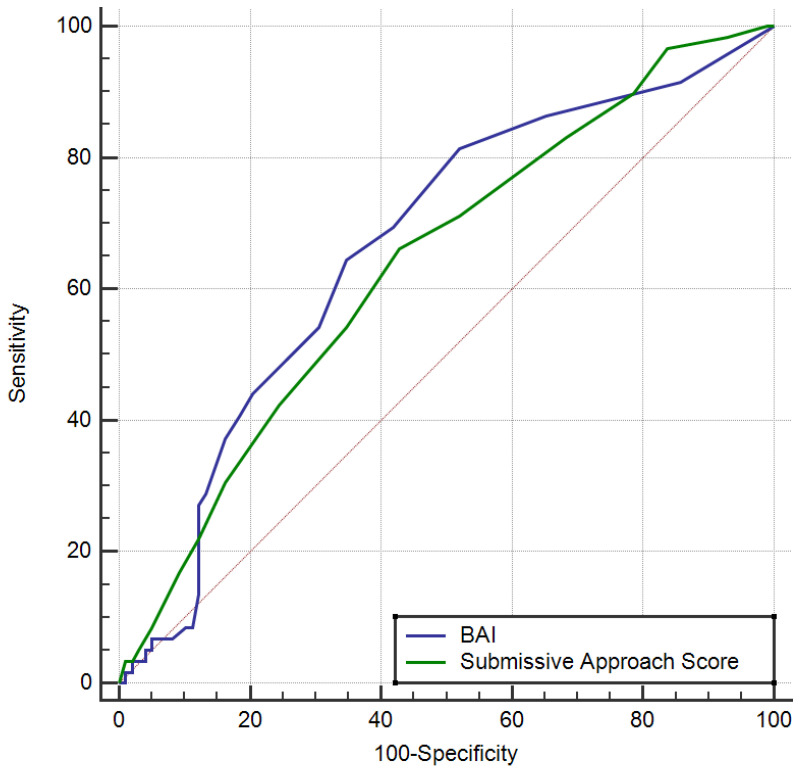
ROC curves of Beck Anxiety Inventory (blue line) and Submissive Approach Score (green line) for predicting fetal anomaly. The diagonal dashed line represents the reference line (AUC = 0.5), indicating random classification. (ROC: receiver operating characteristic; BAI: Beck Anxiety Inventory).

ROC analysis was used to investigate the association between the BAI and fetal anomalies in the Submissive Approach subscale of the CSS, and cut-off values were determined. The cut-off value of 4 for the BAI showed a sensitivity of 64.4% and a specificity of 65.3%. The cut-off value of 6 for the Submissive Approach subscale of the CSS showed a sensitivity of 66.1% and a specificity of 57.1%, indicating that it was also associated (Table 3, Figure 1).

In the patient group, a significant negative correlation was found between spousal support and the BDI, the number of pregnancies, and gravida. A significant positive correlation was found between the BAI and BDI, while significant negative correlations were observed between the BAI and these CSS subscales: the Self-Confident Approach, Seeking of Social Support, and the Optimistic Approach. A significant positive correlation was observed between the BDI and the Helpless Approach subdimension of the CSS, while significant negative correlations were found between the BDI and the Self-Confident Approach and Optimistic Approach subdimensions as well as the gestational age at which the fetal anomaly was detected. A significant positive relationship was found between the Self-Confident Approach and Optimistic Approach subscales. Significant positive relationships were also identified between the Optimistic Approach and Submissive Approach subscales and between the Helpless Approach and Submissive Approach subscales (Table 4).

The overall goodness-of-fit of the logistic regression model was statistically significant based on the Omnibus Test of Model Coefficients (χ² = 57.277, df = 5, *p* < 0.001). The model’s explanatory power was calculated as Cox and Snell R² = 0.306 and Nagelkerke R² = 0.417. The Hosmer–Lemeshow test indicated a good fit between the model and the observed data (*p* = 0.643). A multicollinearity assessment revealed that all independent variables had Variance Inflation Factor (VIF) values ranging between 1 and 1.2, suggesting no significant multicollinearity problems within the model (Table 5).

## 6. Discussion

We compared the anxiety and depression levels, the coping strategies for stress, and the levels of spousal support experienced by pregnant women diagnosed with fetal anomalies during their pregnancies with those of healthy pregnant women with normal pregnancies. We observed higher levels of anxiety and greater use of submissive coping strategies in the group of women diagnosed with fetal anomalies. We found that lower educational levels and living in urban areas were associated with higher frequency of fetal anomaly diagnoses. We observed an increase in the depression levels in the pregnant women as spousal support decreased, and a diagnosis of fetal anomaly was made in the later stages of pregnancy. We found that the use of optimistic, self-confident, and social support-seeking coping strategies by women diagnosed with fetal anomalies helped to reduce their anxiety levels. On the other hand, the use of helpless coping strategies was associated with increased depressive symptoms, while optimistic and self-confident coping strategies were linked to decreases in depressive symptoms.

Pregnancy is an extraordinary process during which physical and hormonal changes occur simultaneously [29]. Negative experiences may affect the mental health of pregnant women, as they are particularly vulnerable to stress [30]. A diagnosis of fetal anomaly can range from minor malformations to the loss of the fetus, and the communication of this diagnosis to the pregnant woman may cause emotional changes [31]. Aite et al. reported that a diagnosis of fetal anomaly is a highly traumatic event for parents expecting a healthy baby, often leading to feelings of anger, depression, and anxiety [32]. Pregnant women who choose to continue their pregnancies after receiving a fetal anomaly diagnosis may experience anxiety and depression [33], while those who decide to terminate their pregnancies may face anxiety, unhappiness, and post-traumatic stress disorder symptoms [34]. Theroux et al. highlighted that pregnant women dealing with fatal anomalies are at risk of developing anxiety, perinatal depression, and post-traumatic stress [35]. Another study found that women who chose to terminate their pregnancies after a fetal anomaly diagnosis had a higher risk of developing depression compared with those who continued their pregnancies [36]. In a study of 131 pregnant women diagnosed with fetal anomalies in the second trimester, most women exhibited normal levels of depression and anxiety and social support was found to reduce existing stress, similar to our study [37]. Although we observed that the women diagnosed with fetal anomalies had higher levels of anxiety, the depression scores were similar, albeit low, between the two groups. The lack of a difference in the depression scores may be due to the short time interval between diagnosis and evaluation. Like in many studies reported in the literature, women who found out that their babies had anomaly/anomalies in the second trimester were more anxious. The lower depression scores in these women may be associated with the severity of the anomaly in the babies of the mothers who were recently diagnosed or with the pregnant women’s use of submissiveness as a method of coping with stress. It is thought that acceptance may facilitate psychosocial stress and serve as a protective mechanism against depressive states. The coping styles adopted by pregnant women may affect their physical and mental health during the perinatal period [38]. Pregnant women have been more likely to use emotion-focused coping strategies to cope with stress [39]. A 2024 study examining stress-coping styles in 301 pregnant women found that 52.8% of the women experienced anxiety and 37.2% showed signs of depression. Emotion-focused coping was positively correlated with depression, anxiety, and stress, while problem-focused coping was negatively associated with these conditions [40]. An investigation in Iran found that pregnant women with fetal anomalies attempted to reduce their stress using various coping strategies, including religiosity, cognitive avoidance, information seeking, and social support [41]. A review also suggested that coping strategies such as problem-solving, acceptance, religiosity, avoidance, and daydreaming have been used by women to cope with the stress caused by a fetal anomaly diagnosis [42]. We observed that women diagnosed with fetal anomalies mostly used the submissive coping strategies and that other coping strategies, except for helpless coping, had a positive impact on the psychological state of the pregnant woman. We believe that factors such as the severity of the anomaly, personality traits, and cultural values could influence the outcome of coping with the stress of a fetal anomaly diagnosis. However, as these factors were not assessed in our study, further research with larger sample sizes is needed to clarify this matter.

As in all other circumstances, social support also plays a crucial role in helping individuals cope with challenges during pregnancy [43]. During and after pregnancy, the closest support is expected to come from spouses, and it is obvious that spousal support is important for maternal mental health [44]. Research has shown that partner-provided support significantly reduces the likelihood of mental health issues during both the prenatal and postnatal periods, and women with lower partner support are more likely to experience perinatal mental health problems [45]. In our study, we found that the women diagnosed with fetal anomalies had similar levels of spousal support to those with healthy pregnancies and that this spousal support helped reduce the depression levels in the women with fetal anomalies. In this study, we also observed that higher scores on the Spousal Support Scale, which evaluated fathers’ support, suggested that the fathers were supportive of the pregnant women, regardless of whether the fetus was healthy or had malformations.

While this is a positive outcome, we believe that multicenter studies that take cultural differences into account will provide clearer results.

We observed that women with lower educational levels and those living in urban areas were more likely to receive fetal anomaly diagnoses. Education level and place of residence are important factors in terms of awareness of health status and ease of access to medical centers. Women with lower educational levels may face higher risks of fetal anomalies due to factors such as poor nutrition and consanguinity [46]. It may be easier for pregnant women living in cities to receive diagnoses of anomalies due to the ease of accessing obstetric care, while in rural areas, anomalies may remain undetected during pregnancy [47].

Among the limitations of our study are that it was a single-center and cross-sectional study; used scales with potential for self-reporting and social desirability bias; failed to exclude potential confounding factors such as the severity and type of fetal anomalies, previous psychiatric history, or use of psychotropic medication; and had a relatively small sample size. The participants were assessed one week after diagnosis to reduce acute emotional bias; however, this short timeframe may have influenced the results. In particular, the lack of significant differences in the depressive symptoms shows that a longer period may be needed for these symptoms to emerge. Another limitation of this study is that the anomalies analyzed ranged in severity—from mild anomalies that could be corrected after birth to severe anomalies, such as trisomy, with recommendations for termination of pregnancy. The fact that our study was conducted in a single center limited the understanding of the cultural context and the emotional impact of prenatal diagnoses. The severity of a fetal anomaly is an important factor that affects the mental state of the mother and should be taken into account in future studies.

## 7. Conclusions

Recognizing the mental health statuses of pregnant women diagnosed with fetal anomalies and providing necessary psychosocial interventions are important to ensuring the health of both mother and baby. Early diagnosis, as in all diseases, is critical for the physical and psychological health of pregnant women. Our results are exploratory, but we believe they can guide further studies. We believe that this study, which aimed to identify psychological conditions caused by fetal anomaly diagnosis during pregnancy, will shed light on future multicenter and large-scale research. A longitudinal follow-up would provide more robust data on the psychological trajectory of these women; thus, in the future, a longitudinal study design will be needed to evaluate the temporal development of psychological symptoms and coping mechanisms and to elucidate the causal or mediating factors in order to better understand the psychological state of expectant mothers diagnosed with fetal anomalies.

## Figures and Tables

**Table 1:** Comparison of all group characteristics.

		Cases (*n* = 59)		Controls (*n* = 98)		*p*
		n	%	n	%	
Age, mean ± SD		29.5 ± 5.5		29.1 ± 4.9		0.587 *
Marital status	Single	57	96,6	98	100.0	0.140 **
	Married	2	3.4	0	0.0	
Education level	High school or below	46	78.0	54	55.1	0.004 **
	University	13	22.0	44	44.9	
Residence	Countryside	9	15.3	31	31.6	0.023 **
	City	50	84.7	67	68.4	
Financial situation	Low	3	5.1	7	7.1	0.911 **
	Moderate	54	91.5	88	89.8	
	High	2	3.4	3	3.1	
Working status	Working	14	23.7	28	28.6	0.507 **
	Not working	45	76.3	70	71.4	
Smoking	Yes	11	18.6	15	15.3	0.586 **
	No	48	81.4	83	84.7	
Specialty in the history of the patient	Yes	1	1.7	3	3.1	0.599 **
	No	58	98.3	95	96.9	
Specialty in the history of the patient’s family	Yes	2	3.4	3	3.1	0.910 **
	No	57	96.6	95	96.9	
Consanguineous marriage	Yes	12	20.3	11	11.2	0.118 **
	No	47	79.7	87	88.8	
Gravidity, median (IQR)		2.0 (1.0–3.0)		2.0 (1.0–3.0)		0.075 ***
Parity, median (IQR)		1.0 (0.0–2.0)		1.0 (0.0–1.0)		0.075 ***
Obstetric visits	Regular	56	94.9	98	100.0	0.051 **
	Irregular	3	5.1	0	0.0	
History of fetal anomaly in previous pregnancies	Yes	6	10.2	2	2.0	0.053 **
	No	53	89.8	96	98.0	
Type of conception	Spontaneous	57	96.6	98	100.0	0.140 **
	IVF	2	3.4	0	0.0	
Gestational age at which fetal anomaly was detected (weeks)	0–12 weeks	6	10.2			-
	12–18 weeks	13	22.0			
	19–24 weeks	29	49.2			
	>24 weeks	11	18.6			
Number of detected anomalies	1	44	74.6	-		-
	2	8	13.6			
	>2	7	11.9			
Currently disabled child	Yes	3	5.1	-		-
	No	56	94.9			
The patient’s thoughts on how the anomaly will affect the baby	Physical	23	40.4	-		-
	Mental	10	17.5			
	Physical + Mental	24	42.1			
Spousal Support Scale, median (IQR)		77.0 (67.0–81.0)		77.5 (74.0–80.0)		0.263 ***
Beck Anxiety Inventory, median (IQR)		6.0 (3.0–12.0)		3.0 (1.0–7.0)		<0.001 ***
Beck Depression Inventory, median (IQR)		8.0 (4.0–11.0)		6.0 (4.0–10.0)		0.122 ***
Self-Confident Approach, median (IQR)		14.0 (12.0–16.0)		14.0 (12.0–17.0)		0.796 ***
Seeking Social Support, median (IQR)		8.0 (6.0–9.0)		8.0 (7.0–9.0)		0.270 ***
Optimistic Approach, median (IQR)		10.0 (8.0–12.0)		10.0 (8.0–12.0)		0.460 ***
Helpless Approach, median (IQR)		9.0 (7.0–12.0)		8.0 (5.0–12.0)		0.218 ***
Submissive Approach, median (IQR)		8.0 (5.0–10.0)		6.0 (4.0–8.0)		0.004 ***

* Student’s *t*-test, ** Chi-square analysis, *** Mann–Whitney U test. *n*, number of samples; SD, standard deviation; IVF, in vitro fertilization.

**Table 2:** Logistic regression analysis of fetal anomaly diagnosis.

	B	S.E.	*p*	OR (95% CI)
Education Level (Ref = University)	1.177	0.477	0.014	3.243 (1.274–8.254)
Residence (Ref = Countryside)	1.117	0.526	0.034	3.054 (1.090–8.560)
Gestational Age (Ref = 2. Trimester)	−22.207	0.857	0.998	0.000 (0.000–)
Submissive Approach	0.078	0.057	0.170	1.081 (0.967–1.209)

Ref, reference; B, regression coefficient; S.E., standard error; OR, odds ratio.

**Table 3:** Specificity and sensitivity of measured parameters in determining fetal anomaly.

	Space	*p*	95% Confidence Interval		Sensitivity	Specificity	PPV	NPV
			Lower Limit	Upper Limit				
Beck Anxiety Inventory > 4	0.665	<0.001	0.586	0.739	64.4	65.3	52.8	75.3
Submissive Approach > 6	0.637	0.002	0.557	0.713	66.1	57.1	48.1	73.7

PPV, positive predictive value; NPV, negative predictive value.

**Table 4:** Pairwise correlations between the scale scores in the case group.

		Spousal Support Scale	BAI	BDI	Self-Confident Approach	Seeking Social Support	Optimistic Approach	Helpless Approach	Submissive Approach
Beck Anxiety Inventory	r	−0.187							
	*p*	0.157							
Beck Depression Inventory	r	−0.409	0.366						
	*p*	0.001	0.005						
Self- Confident Approach	r	0.200	−0.289	−0.308					
	*p*	0.128	0.026	0.019					
Seeking Social Support	r	0.124	−0.261	−0.246	0.219				
	*p*	0.350	0.046	0.063	0.096				
Optimistic Approach	r	0.186	−0.295	−0.309	0.630	−0.053			
	*p*	0.159	0.023	0.018	0.000	0.688			
Helpless Approach	r	−0.178	0.178	0.311	0.052	−0.199	0.055		
	*p*	0.177	0.178	0.017	0.694	0.131	0.679		
Submissive Approach	r	−0.020	0.073	−0.048	0.211	0.012	0.278	0.423	
	*p*	0.881	0.584	0.723	0.108	0.931	0.033	0.001	
Age	r	0.023	−0.054	0.121	−0.073	0.126	−0.103	−0.008	0.082
	*p*	0.863	0.689	0.370	0.589	0.346	0.443	0.953	0.539
Gravidity	r	−0.287	0.188	0.121	−0.126	0.011	−0.255	−0.026	0.087
	*p*	0.028	0.154	0.366	0.344	0.936	0.051	0.845	0.514
Parity	r	−0.145	0.168	0.065	−0.064	0.025	−0.215	0.070	0.132
	*p*	0.274	0.203	0.628	0.629	0.849	0.102	0.599	0.320
Gestational weeks at which fetal anomaly was detected	r	0.074	0.017	−0.263	−0.001	0.120	0.051	−0.003	0.067
	*p*	0.578	0.896	0.046	0.993	0.365	0.701	0.984	0.616
Number of detected anomalies	r	0.090	0.012	0.126	−0.042	0.097	0.003	0.084	0.200
	*p*	0.500	0.926	0.346	0.750	0.465	0.982	0.526	0.129

(BAI, Beck Anxiety Inventory; BDI, Beck Depression Inventory).

**Table 5:** Goodness-of-fit and multicollinearity assessment of the logistic regression model.

Model Characteristics	Value
Omnibus Test of Model Coefficients	χ² = 57.277, df = 5, *p* < 0.001
−2 Log Likelihood	150.582
Cox and Snell R²	0.306
Nagelkerke R²	0.417
Hosmer–Lemeshow Test	χ² = 6.034, df = 8, *p* = 0.643
Mean VIF	1.075

## Data Availability

The original contributions presented in this study are included in the article. Further inquiries can be directed to the corresponding author.
